# Image-guided left ventricular lead placement in cardiac resynchronization therapy for patients with heart failure: a meta-analysis

**DOI:** 10.1186/s12872-015-0034-0

**Published:** 2015-05-10

**Authors:** Yan Jin, Qi Zhang, Jia-liang Mao, Ben He

**Affiliations:** Department of Cardiology, Ren Ji hospital, School of Medicine, Shanghai Jiao Tong University, 200001 Shanghai, China

**Keywords:** Cardiac resynchronization therapy, Heart failure, Echocardiography, Image-guided, Echocardiography guided

## Abstract

**Background:**

Heart failure (HF) is a debilitating condition that affects millions of people worldwide. One means of treating HF is cardiac resynchronization therapy (CRT). Recently, several studies have examined the use of echocardiography (ECHO) in the optimization of left ventricular (LV) lead placement to increase the response to CRT. The objective of this study was to synthesize the available data on the comparative efficacy of image-guided and standard CRT.

**Methods:**

We searched the PubMed, Cochrane, Embase, and ISI Web of Knowledge databases through April 2014 with the following combinations of search terms: left ventricular lead placement, cardiac resynchronization therapy, image-guided, and echocardiography-guided. Studies meeting all of the inclusion criteria and none of the exclusion criteria were eligible for inclusion. The primary outcome measures were CRT response rate, change in LV ejection fraction (LVEF), and change in LV end systolic volume (LVESV). Secondary outcomes included the rates of all-cause mortality and HF-related hospitalization.

**Results:**

Our search identified 103 articles, 3 of which were included in the analysis. In total, 270 patients were randomized to the image-guided CRT and 241, to the standard CRT. The pooled estimates showed a significant benefit for image-guided CRT (CRT response: OR, 2.098, 95 % CI, 1.432–3.072; LVEF: difference in means, 3.457, 95 % CI, 1.910–5.005; LVESV: difference in means, −20.36, 95 % CI, −27.819 – −12.902).

**Conclusions:**

Image-guided CRT produced significantly better clinical outcomes than the standard CRT. Additional trials are warranted to validate the use of imaging in the prospective optimization of CRT.

## Background

Heart failure (HF) is a debilitating and highly prevalent condition. Recent estimates of the prevalence of heart failure in Asia range from almost 1 % to greater than 6.5 %, [1, 2], while it ranges from 2 % to 3 % in the United States [3], and in two of the representative European populations [4, 5]. The World Health Organization’s most recent report on disease burden indicated that almost 6 million people were diagnosed with HF in a single year [6]. Many of these were elderly, and multiple studies have demonstrated that the prevalence of HF is greater in older individuals [3, 7]. The ageing of the world’s population translates to an even greater burden in the coming decades [1, 8–10]. Moreover, the increasing prevalence of diabetes, a risk factor and co-morbidity of HF, further worsens the clinical outcomes [11–22]. The management of HF may need to focus not only on prevention, but also on increasing the treatment success: one recent study of approximately 5 million people in Scotland found that from 1986 to 2003, the median survival after first hospitalization only increased to 1.79 years for women and 2.34 years for men (up from 1.32 and 1.33 years, respectively) [23].

Among those with HF, arrhythmias, and dyssynchrony are common abnormalities. One treatment option for these patients is cardiac resynchronization therapy (CRT). Since its implementation over 20 years ago, CRT has been proven to improve the outcomes of those with HF [24–28]. However, almost one third of patients who receive CRT do not benefit, giving rise to the concept of non-response [29, 30]. Although non-response is an ongoing and controversial issue in CRT [24, 31], studies conducted over 10 years ago revealed that optimizing the pacing site can improve the outcomes and response [32–34].

The use of echocardiography (ECHO) is one method by which lead placement may be optimized. Speckle tracking ECHO (STE), which traces patterns of acoustic signals (i.e., speckles) over time, is a validated device to measure myocardial strain. By measuring strain and strain rate, STE can detect regions of scarred myocardium as well as crucial features of dyssynchrony [35]. Vector velocity imaging ECHO (ECHO-VVI) is a variation on STE that also tracks the movement of the user-defined endocardial border [36]. Several groups have used STE or ECHO-VVI not only to define the systolic volume and the latest site of activation, but also to show an association between these factors, concordant lead placement, and CRT response [37–39]. Given the success of these studies, research is now focused on the use of ECHO techniques such as, STE and VVI to prospectively guide lead placement to maximize the effects of CRT. However, a quantitative synthesis of the available data has not yet been published. Accordingly, the objective of the current study was to determine the comparative efficacy and safety of image-guided and conventional CRT.

## Methods

### Search strategy

The methods used in this review and meta-analysis adhere to the current best practices for conducting systematic reviews of the literature. We searched the PubMed, Cochrane, Embase, and ISI Web of Knowledge databases through April 28, 2014 by using the following combinations of search terms: left ventricular lead placement, cardiac resynchronization therapy, imaging-guided, and echocardiographically guided. Our search strategy in PubMed included the following terms: left ventricular lead placement AND cardiac resynchronization therapy AND (imaging OR echocardiographically guided). Our search filters were: abstract available, English language, and human species.

After the removal of duplicate citations, we identified relevant studies using a 2-step search process. In the first step, we screened the title and abstracts of all citations identified in the search against the inclusion and exclusion criteria (see below). In the second step, we retrieved the full texts of the remaining articles. Studies meeting all of the inclusion criteria and none of the exclusion criteria were included in the analysis.

### Selection of studies

The inclusion criteria for this meta-analysis were as follows: 1- The study was a randomized controlled trial (RCT) or a 2-arm prospective study; 2- The study enrolled patients with heart failure who received a CRT pacemaker (CRT-P) or a CRT implantable cardioverter defibrillator (CRT-D) device. A study was excluded for the following reasons: 1- It was a retrospective or cohort study, letter, comment, editorial, or case report; 2- The enrolled patients received other interventions besides those mentioned above.

### Data extraction

The following data were extracted from the included studies: first author, year of publication, study design, trial name if applicable, type of intervention, follow-up time, number of patients enrolled, proportion of male patients, New York Heart Association (NYHA) grade of the enrolled patients, width of the QRS, baseline and post-treatment left ventricular ejection fraction (LVEF), baseline and post-treatment left ventricular end-systolic volume (LVESV), concordance of the LV lead to the latest site of activation, odds ratio (OR) for the CRT response, and the rates of CRT response, all-cause mortality, and HF-related hospitalization. Two independent reviewers extracted the data from the eligible studies, and any disagreements were resolved by consulting a third reviewer.

### Outcome measures

The primary outcome measures of this analysis were CRT response rate, change in LVEF, and change in LVESV. All 3 included studies defined the CRT response, differently. Khan et al. defined response as LV reverse remodeling (≥15 % decrease in LVESV) at 6 months, Saba et al. as LV reverse remodeling or a ≥5 % absolute increase in LVEF, both with no primary endpoint, and Bai et al. required 2 of 3 criteria: LV reverse remodeling, a ≥20 % relative increase in LVEF, or a 1-class decrease in NYHA functional status.

Secondary outcomes included the rates of all-cause mortality and HF-related hospitalization.

### Quality assessment

The quality of the included studies was assessed with the Cochrane Risk of Bias tool, a component of Review Manager 5.1. The assessment is categorized into six domains—random sequence generation (selection bias), allocation concealment (selection bias), blinding of patients and personnel (detection bias), blinding of outcome assessment (detection bias), incomplete outcome data (attrition bias), and selective reporting risk (reporting bias)—that are described in Chapter 8 of the Cochrane Handbook for Systematic Reviews Interventions [40].

### Statistical analysis

The OR and corresponding 95 % confidence intervals (CI) of the CRT response, difference in means of change in LVEF, and the difference in means of change in LVESV were used to evaluate the efficacy of image-guided CRT compared to standard CRT. For the OR of the CRT response, an OR > 1, indicates that the image-guided CRT group tended to have a higher response rate than the standard CRT group, while an OR < 1, indicates that the image-guided CRT group tended to have a lower response rate than the standard CRT group, and an OR = 1, indicates that the response rates were similar for both the groups. For the difference in means of LVEF, an OR > 0, indicates that image-guided CRT lead to a greater change (i.e., from pre- to post-treatment) in outcome than did standard CRT, an OR < 0, indicates that image-guided CRT lead to a lesser change in the outcome than the standard CRT, while an OR = 0, indicates that the two procedures had similar effects on the change in outcome. For the difference in means of LVESF, an OR > 0, indicates that image-guided CRT lead to a lesser change in the outcome than the standard CRT, an OR < 0, indicates that image-guided CRT lead to a greater change in outcome than did the standard CRT, and an OR = 0 indicates that the 2 procedures affected the change in outcome to a similar extent. Either a Cochran Q statistic of P < 0.1 or an I^2^ statistic > 50 % was considered as obvious heterogeneity among studies. When heterogeneity was detected, a random-effects model was preferred; otherwise, a fixed-effects model was considered. Sensitivity analysis was performed by using the leave-one-out approach. A funnel plot and Egger’s test were not used because publication bias cannot be analyzed accurately with 5 studies or less [41]. A two sided *P* value < 0.05 was considered statistically significant. All statistical analyses were performed with Comprehensive Meta-Analysis statistical software, version 2.0 (Biostat, Englewood, NJ, USA).

## Results

### Study selection

**Fig. 1:**
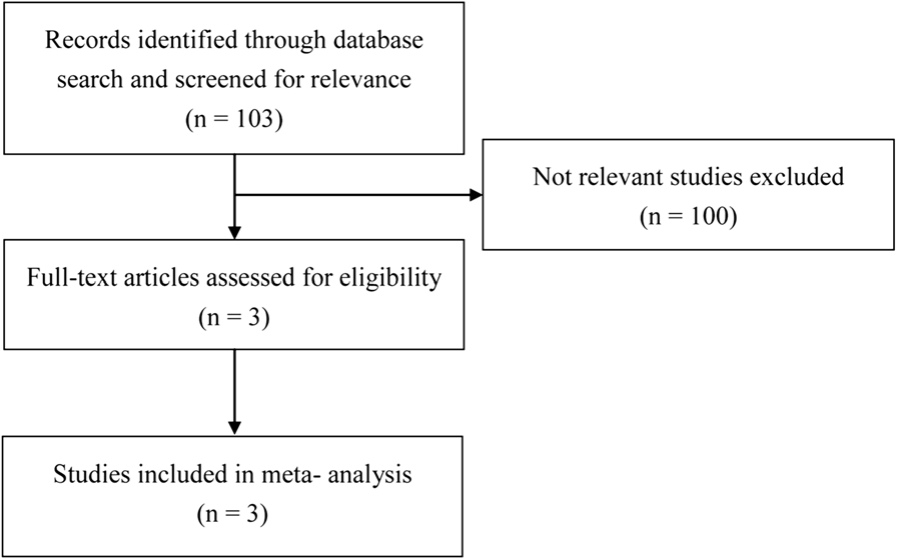
A flow diagram of the process for study selection

A flow chart for study selection is shown in Fig. 1. Of the 103 studies identified in the initial search, 100 were excluded after a full text review, due to the lack of relevance. The remaining 3 studies met all of the inclusion criteria and were included in the meta-analysis.

### Study characteristics

A summary of the characteristics of the trials and patients is presented in Table 1. The included studies consisted of 2 RCTs, by Khan et al. [42] and Saba et al. [43], conducted in the United Kingdom and United States, respectively, and 1 prospective cohort study by Bai et al. [44] conducted in the United States and Italy. The studies by Khan et al. and Saba et al. assessed speckle-tracking ECHO, while Bai et al. assessed ECHO coupled with VVI. The standard CRT used empiric placement of LV leads and was facilitated only by coronary venography, with a preference for a lateral, posterior, or posterolateral region as the implantation site. In total, 511 patients were enrolled, with 270 randomized to the image-guided group and 241 to the standard CRT group. Follow-up times for the studies ranged from 6 months to just over 3 years. The mean or median age of enrolled patients ranged from 61 to 72 years, with 60 % to 80 % of the patients being men. The majority of patients were classified as New York Heart Association (NYHA) functional class III or IV, although Saba and colleagues enrolled a small percentage of patients (approximately 12 %) belonging to class II. The average left ventricular ejection fraction (LVEF) and QRS width were similar among the 3 studies, ranging from 23 % to 26 % and 153 to 162 milliseconds, respectively.

**Table 1:** Summary of basic characteristics of the studies included in the meta-analysis

First author (year)	Study design (trial name)	CRT intervention	Follow-up time	Patient no.	Age (yrs)	Gender, male (%)	NYHA grade	LVEF (%)	QRS width (ms)
Saba (2013) [43]	RCT (STARTER)	Image-guided	1.8 ± 1.3 yrs	110	66 ± 11	70 %	II/III/IV: 16/64/20^a^	26 ± 6	157 ± 27
		Standard^b^		77	67 ± 13	78 %	II/III/IV: 8/71/21^a^	26 ± 7	162 ± 27
Khan (2012) [42]	RCT (TARGET)	Image-guided	2 yrs	110	72 (65–76)	77 %	III/IV: 95/15^c^	23 ± 6	157 (148–170)
		Standard^b^		110	72 (64–80)	80 %	III/IV: 93/17^c^	23 ± 7	159 (146–170)
Bai (2011) [44]	Prospective study	Image-guided	6 mths	50	66 ± 11	60 %	III or IV: 3.10 ± 0.30^d^	23 ± 7	153 ± 23
		Standard^b^		54	64 ± 9	74 %	III or IV: 3.07 ± 0.26^d^	26 ± 6	155 ± 29

*Abbreviations*: *CRT* cardiac resynchronization therapy, *NYHA* New York Heart Association, *LVEF* left ventricular ejection fraction, *RCT* randomized control trials, *yrs* years, *mths* months, *no* number

Data are presented as mean ± SD or median (inter-quartile range), unless noted otherwise. ^b^Standard procedures means that empiric placement of LV leads was only facilitated by coronary venography, with a preference for a lateral, posterior, or posterolateral region as an implantation site. ^a^data are presented as percentage; ^c^data are presented as counts; ^d^data are presented as mean ± SD

### Outcomes of interest

Data for the outcomes of each study are summarized in Table 2. While only Khan et al. and Bai et al., reported the OR for CRT response, all 3 publications reported data for the percentage of subjects who responded to CRT and for the other two primary outcomes of change from the baseline in LVEF and LVESV. Just one study, that of Saba et al., reported group-specific data for the secondary outcomes of the rates of all-cause mortality and HF-related hospitalization.

**Table 2:** Summary of outcomes of the included studies

First author (year)	CRT intervention	Concordance of LV lead to latest site of activation, %	OR for CRT response (95 % CI)	CRT response, count (%)	LVESV (ml), mean ± SD		Change from baseline	LVEF (%), mean ± SD			All-cause mortality, count (%)	HF-related hospitalization, count (%)
					Baseline	Post treatment		Baseline	Post treatment	Change from baseline		
Saba (2013) [43]	Image-guided	Exact/adjacent:	NR	50 (57 %)	140 ± 59	NR	−30 ± 29 (%)^a^	26 ± 6	38 ± 12.8^b^	12 ± 11	15 (13.6 %)	16 (14.5 %)
		30/NR										
		Exact or adjacent: 85										
		Remote: 15										
	Standard	Exact/adjacent:		22 (35 %)	144 ± 63	NR	−20 ± 25 (%)^a^	26 ± 7	35 ± 11.45^b^	9 ± 10	15 (19.5 %)	21 (27.3 %)
		12/NR										
		Exact or adjacent: 66										
		Remote: 33										
Khan (2012) [42]	Image-guided	Exact/adjacent:	1.92 (1.08, 3.39)	72 (70 %)	157 ± 56	111 ± 43	−46 ± 33 (ml)	23 ± 6	31 ± 9	8 ± 7	22 (10 %)	18 (8 %)
		63/26										
		Exact or adjacent: NR										
		Remote: 10										
	Standard	Exact/adjacent:	1.0 (reference)	57 (55 %)	154 ± 52	128 ± 50	−26 ± 23 (ml)	23 ± 7	28 ± 10	5 ± 8		
		47/29										
		Exact or adjacent: NR										
		Remote: 25										
Bai (2011) [44]	Image-guided	NR	2.68 (1.08-6.65)^c^	41 (82 %)	172 ± 65	129 ± 65	−43 ± 65 (ml)	23 ± 7	34 ± 10	11 ± 8.89^d^	NR	NR
	Standard	NR	1.0 (reference)	34 (63 %)	159 ± 74	141 ± 82	−18 ± 78 (ml)	26 ± 6	32 ± 9	6 ± 7.94^d^	NR	NR

*Abbreviations*: *CRT* cardiac resynchronization therapy, *LV* left ventricular, *EF* ejection fraction, *LVESV* left ventricular end-systolic volume, *ml* milliliters, *SD* standard deviation, *NR* not reported, *HF* heart failure

^a^Represented as a relative change before and after treatment

^b^Data were estimated according to the reported results of baseline and change from baseline; n = 87 for the image-guided group and n = 62 for the standard group

^c^With adjustment for clinical characteristics

^d^Data were estimated according to the reported LVEF at baseline and post-treatment

#### CRT response

**Fig. 2:**
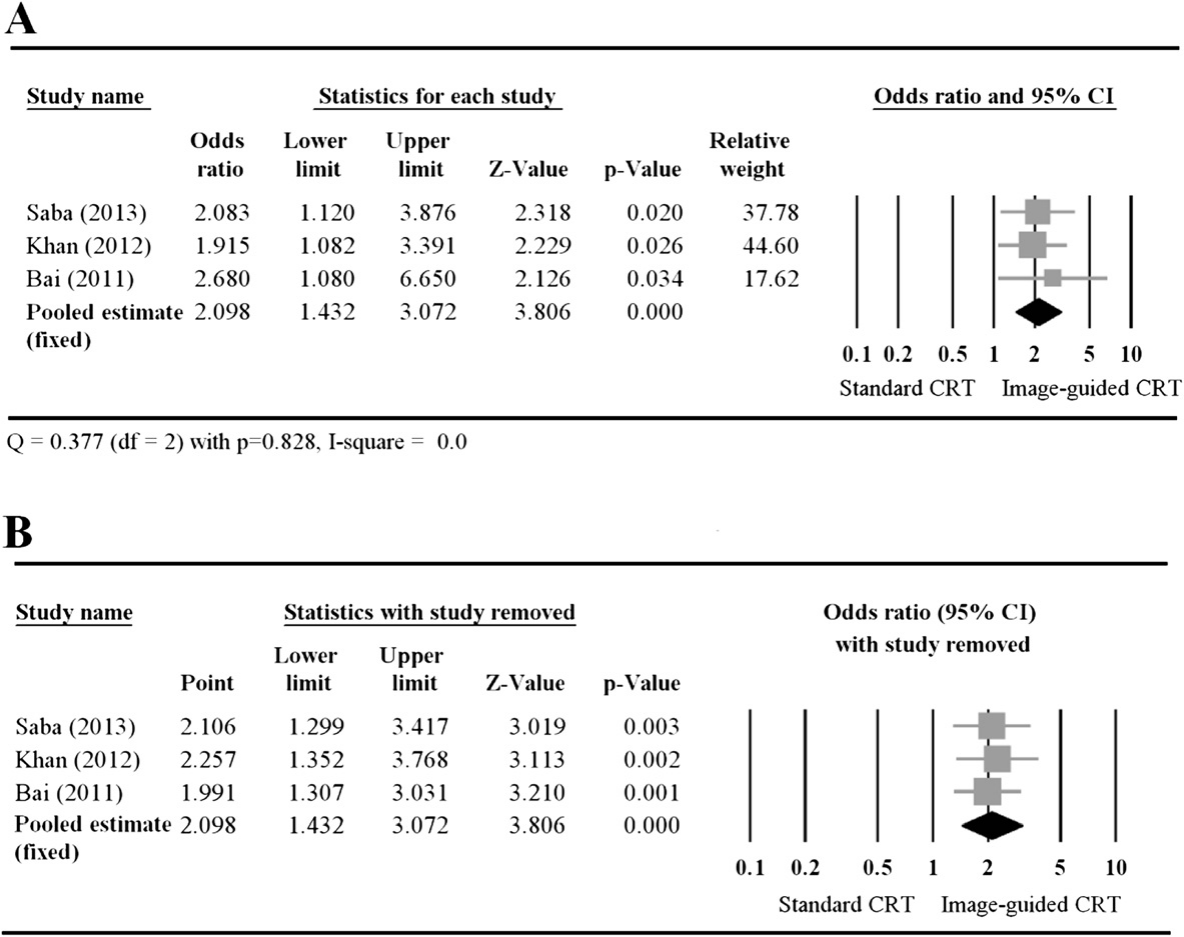
A Forest plot showing the results of a meta-analysis of CRT response rate (**A**) and the corresponding sensitivity-analysis (**B**) for the patients who received image-guided or standard CRT. (**A**) The pooled estimate favors image-guided CRT. (**B**) The pooled estimates of all 3 studies and each pair are similar. Abbreviations: CI, confidence interval; CRT, cardiac resynchronization therapy; Lower limit, lower bound of the 95 % CI; Upper limit, upper bound of the 95 % CI

We generated a Forest plot (Fig. 2A) to illustrate the individual and pooled ORs for CRT response. All 3 groups reported that patients undergoing image-guided CRT were more likely to have a higher CRT response. For the pooled estimate, the Cochran Q statistic was 0.377 with *p* = 0.828 and the I^2^, was 0. These values indicated a lack of obvious heterogeneity among the studies; thus, a fixed-effect model was performed to determine the pooled OR for CRT. The pooled estimate for the OR of CRT response also showed that patients in the image-guided CRT treatment group had a significantly higher response rate than those who received standard CRT (OR: 2.098; 95 % CI: 1.432–3.072, *p* < 0.001). A sensitivity analysis based on the leave-one-out approach (Fig. 2B) determined that the pooled OR was robust, because the ORs determined with each study removed were similar to that of all 3 studies combined.

#### Change in LVEF

**Fig. 3:**
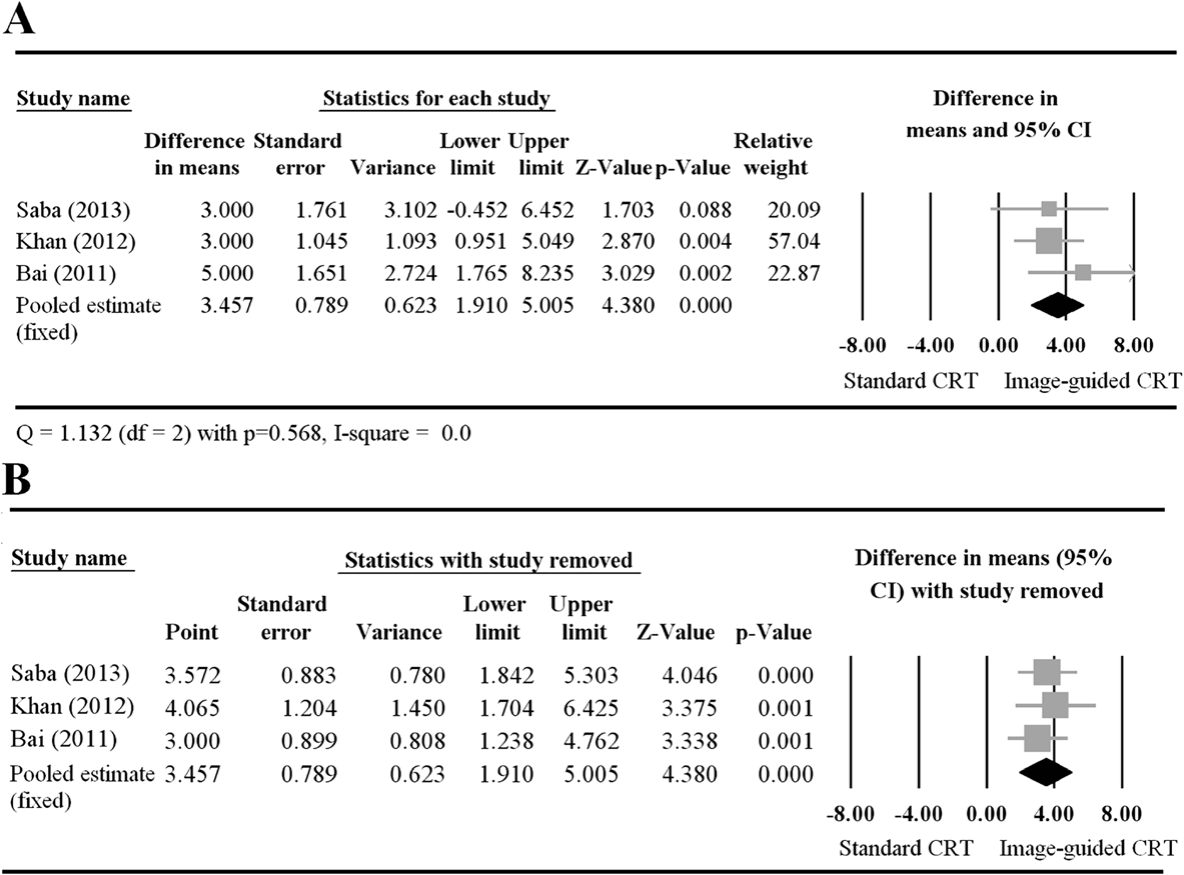
A Forest plot showing the results of a meta-analysis of post-treatment change in LVEF (**A**) and the corresponding sensitivity-analysis (**B**) for the patients who received image-guided or standard CRT. (**A**) The pooled estimate favors image-guided CRT. (**B**) The pooled estimates of all 3 studies and each pair are similar. Abbreviations: CI, confidence interval; CRT, cardiac resynchronization therapy; LVEF, left ventricular ejection fraction; Lower limit, lower bound of the 95 % CI; Upper limit, upper bound of the 95 % CI

The individual and combined differences in means for change in LVEF are illustrated in a Forest plot in Fig. 3A. Khan et al. and Bai et al. both reported that the change in LVEF was significantly greater for the image-guided CRT treatment group than for the standard CRT group. The results of Saba et al. also favored image-guided CRT, but lacked statistical significance. As with the outcome of CRT response, the studies lacked heterogeneity (Cochran Q = 1.132, *p* = 0.568; I^2^ = 0); therefore, a fixed-effects model was performed. The pooled estimate of the difference in means of the change in LVEF was 3.457 (95 % CI: 1.910–5.005, *p* < 0.001), which indicates that patients who underwent image-guided CRT experienced a significantly greater improvement in LVEF than did those who underwent standard CRT. Our sensitivity analysis revealed that the pooled estimate of the difference in means for all 3 studies was similar to those for each pair (Fig. 3B).

#### Change in LVESV

**Fig. 4:**
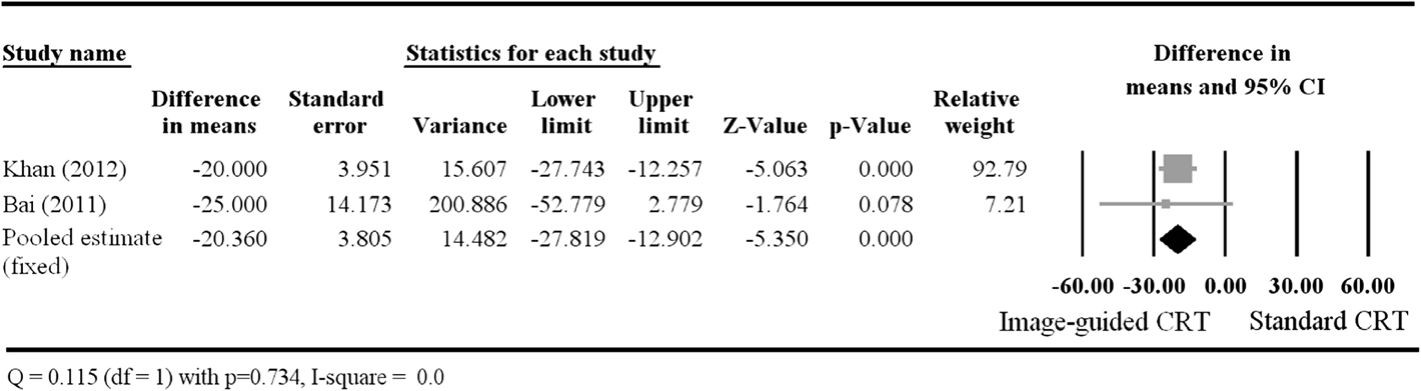
A Forest plot showing the results of a meta-analysis of post-treatment change in LVESV (in milliliters) for the patients who received image-guided or standard CRT. The pooled estimate favors image-guided CRT. Abbreviations: CI, confidence interval; CRT, cardiac resynchronization therapy; LVESV, left ventricular end-systolic volume; Lower limit, lower bound of the 95 % CI; Upper limit, upper bound of the 95 % CI

The Forest plot in Fig. 4 shows the individual and pooled difference in means for change in LVESV. All of the studies found that the improvement in LVESV was greater in the image-guided CRT group than in the standard therapy group; however, the data of Bai et al. did not reach statistical significance. Because Saba et al. reported change as a relative value, data from this trial were not included in the meta-analysis. As with the other primary outcomes, a fixed-effects model was performed, due to the lack of heterogeneity (Cochran Q = 0.115, *p* = 0.734; I^2^ = 0). The pooled estimate of the difference in means of the change in LVESV (−20.36, 95 % CI: −27.819 – −12.902, *p* < 0.001) showed that the average reduction of LVESV was greater in the image-guided CRT group than in the standard CRT group.

#### Secondary and other outcomes

Reporting of the secondary outcomes of this meta-analysis—all-cause mortality and HF-related hospitalization—varied widely among the included studies. Saba et al. reported group-specific data for both outcomes, while Khan et al. reported the data for both outcomes for all enrolled patients. However, Bai et al. did not report data for either of the outcomes. The results of Saba and colleagues indicate that mortality from any cause may be similar for both the treatments, but use of image-guided CRT may lead to lesser HF-related hospitalizations.

We also compared data reported by Khan et al. and Saba et al. for the concordance of LV lead placement to the latest site of activation (Table 2; Bai et al. did not report this outcome). In both trials, exact or adjacent concordance was obtained for a greater number of patients in the image-guided group than in the standard CRT group. Accordingly, the image-guided CRT group had a lower percentage of remote placements, with remote placements being more than twice as numerous in the standard CRT group.

### Quality assessment

We used the Risk of Bias tool, a component of Cochrane’s Review Manager, to assess the quality of the included studies (Table 3). Our analysis revealed different levels of quality in the domains of selection, performance, and detection bias (see Methods). The publication by Khan et al. clearly documented the utility of a computer-generated system and stated that the group assignment used a central, fully independent system. Moreover, the patients and the assessor were blinded, and the process of blinding was clearly stated. Therefore, this trial had a low risk of bias in these domains. Saba et al. stated that the patients and the healthcare providers were blinded, but the implanting physician was not. In addition, these authors presented insufficient information about the stratification methods, resulting in an unclear risk of bias. The study of Bai et al. had a high risk of selection bias, as it was not randomized and allocation concealment was not described in the published results. However, the authors confirmed that consecutive patients were enrolled and that local cardiologists were blinded to the procedure. In addition, they reported that the operators performed at least 10 image-guided CRT implants before including patients, making it unlikely that bias could occur as a result of non-randomization or sequential learning. All 3 publications contained information on the follow-up time and the methods used to handle missing data, and thus the studies had a low risk of attrition bias. They also had a low risk of reporting bias, as data were presented for all of the pre-specified outcomes.

**Table 3:** Quality assessment of the included studies

First author (year)	Selection bias (random sequence generation)	Selection bias (allocation concealment)	Performance bias (blinding of participants and personnel)	Detection bias (blinding of outcome assessment)	Attrition bias (incomplete outcome data)	Reporting bias (selective reporting)
Saba (2013) [43]	Y	Unclear	Y	Unclear	Y	Y
Khan (2012) [42]	Y	Y	Y	Y	Y	Y
Bai (2011) [44]	N	N	Unclear	Y	Y	Y

*Y* low risk of bias, *N* high risk of bias, *Unclear* insufficient data for judgment

## Discussion

In this meta-analysis, we determined the comparative efficacy of image-guided and standard CRT in patients with HF. Three studies enrolling approximately 500 patients were included in the analysis. Although other meta-analyses have addressed the ability of STE and/or VVI to predict or identify the determinants of CRT response, we believe that ours is one of the only analyses published to date that examines the ability of these procedures to prospectively optimize lead placement. Accordingly, this work provides an important update to the field. The results indicate that image-guided CRT is significantly better than the standard CRT in improving the overall response, LVEF, and LVESV. A better CRT response is consistent with better improvement of LVEF and LVESV in the image-guided group, as compared to the standard group. A meta-analytic approach highlights the need to synthesize data from multiple studies, as some of the individual data lacked statistical significance. This analysis also revealed the variation in endpoints reported by RCTs or prospective studies of CRT and leads us to propose that greater standardization will increase the benefits of future research. This is especially true for HF-related hospitalization, as these data could provide crucial information on the financial burden of HF.

The effect estimates determined by the pooled analysis were largely concordant with each study’s individual result. The diverse definitions of the CRT response in the studies included, may have contributed to the wide range of response rates (control group: 22 %–57 %, image-guided group: 41 %–72 %). The data reported for the change in LVEF, while supporting image-guided CRT, did not reach statistical significance in one of the three studies. Our pooled estimate was able to take advantage of the increased patient number to determine whether the use of STE or VVI in guiding the LV lead placement has a significant effect on this clinical outcome. Similarly, when data for the change in LVESV were pooled, we found a significant benefit for patients in the image-guided group.

Although we did not conduct a meta-analysis of data for the concordance of LV lead position to the site of latest activation, our summarized results support the hypothesis that the superior outcome in patients in the image-guided CRT group, is in part due to the improved concordance of lead placement. Both the TARGET and STARTER trials found that patients whose LV leads were exactly concordant or adjacent had significantly better outcomes than patients with remote leads. These results were confirmed by 3 recent studies: Kydd et al., analyzed a subset of patients from the derivation and randomized groups of the TARGET trial, [45]. They have demonstrated a superior CRT response and improved survival by optimal LV lead position at the site of latest mechanical activation, and by avoiding low strain amplitude (scar). Similar results were reported by Marek et al. in a subgroup of the STARTER population of patients without left bundle branch block (LBBB) or a QRS duration between 120 and 149 milliseconds [46], and by Adelstein et al., where they observed defibrillator therapy-free survival at 3 years among the patients in STARTER trial [47]. Though, our present data is not sufficient enough to perform a meta-analysis of concordant/adjacent vs. remote LV lead, the aforementioned studies strongly suggest the favorable impact of image-guided CRT on long term survival.

Image-guided CRT may also provide superior results by virtue of its ability to target LV leads to sites that are remote from regions with low radial strain, a hallmark of scarred myocardium. The TARGET trial assessed the relationship between sites of scar and lead placement, and discovered that the lead placement in regions remote from a scar yields improved outcomes. Sade et al. addressed the issue of scars in a follow-up to the STARTER trial [48]. This study evaluated both the effects of lead placement within the scarred segments and the interaction between scars and activation. In ischemic patients, LV lead placement within or adjacent to the scarred segments had a significantly negative effect on survival when compared to leads that were remote to the scar. Moreover, the clinical outcomes were better in patients when the lead was placed at the latest site of activation and remote to a scar, than in those with a lead placed both within a scar and at the latest-activation site. As Vernooy et al. recently noted [49], the current body of research strongly suggests that the presence of scars near a lead will hinder the response to CRT. The results from the STARTER and TARGET trials, as well as those from a recent study by Kydd et al. [50], support this conclusion. However, Vernooy and colleagues noted the lack of evidence to recommend targeting LV leads during CRT. The work of Sade et al. demonstrated the additive value of this aspect of optimization, as survival of patients with a lead being both concordant and remote to the scar was better than a non-concordant lead being remote to a scar [48]. Taken together, the available data from the 3 patient populations included in our analysis indicate that the use of image-guided CRT will improve outcomes, as it provides superior ability not only to detect and avoid scar, but also to place leads in a location best suited for resynchronization.

The studies included in our meta-analysis showed variability in both the primary and secondary endpoints. Bai et al. and Khan et al. had a primary endpoint of response, but the primary endpoint of Saba et al.—all-cause mortality or HF-related hospitalization—was a secondary outcome only for Khan et al. Among secondary endpoints, just Khan et al. and Bai et al. assessed post-procedural NYHA functional class. The included studies also varied in the reporting of regression analyses to determine factors with predictive value: 2 used regression analyses to identify predictors of outcome; however, the outcomes differed: Bai et al. reported predictors of CRT response while Khan et al. identified predictors of LV reverse remodeling. These differences limit the strength of the present meta-analysis and prompt us to suggest an increased uniformity in endpoint classification across trials. Likewise, whether the efficacy and effectiveness of image-guided CRT response observed in this study will hold true irrespective of the underlying heart failure etiology is not known, as the studies did not compare the CRT response in patients with non-ischemic and ischemic heart failure. Nevertheless, studies elsewhere suggest that, though the improvement of the left ventricular function and remodeling is greater in non-ischemic cardiomyopathy patients, there was no difference in CRT response in terms of reduced mortality or heart failure hospitalization rate between ischemic and non-ischemic heart failure patients [51]. An increase in LV outflow-tract velocity-time-integral and LV ejection fraction after simultaneous CRT was greater in non-ischemic heart failure patients. However, interventricular pacing interval optimization yielded a similar CRT response in both ischemic and non-ischemic heart failure patients [52].

This study had several limitations. First, the relatively small sample size of the three studies that met the eligibility criteria, along with the differences in clinical outcomes analyzed, is a major limitation of the current meta-analysis. Although the pooled estimates were robust in our analysis, similar results from a larger cohort of patients would further strengthen our conclusions. However, reports indicate that systematic reviews with small numbers of included studies will be accurate in the final point estimates in the long run [53]. Second, we could not conduct a meta-analysis of clinical outcomes such as HF-related hospitalization and mortality because of insufficient data, as mentioned above. Third, we did not conduct a subgroup analysis of the comparative performance of image-guided and standard CRT to place leads away from regions of scar.

## Conclusion

In conclusion, we have conducted a meta-analysis of the comparative efficacy of image-guided and standard CRT in patients with HF. Our results indicate that a strategy of echocardiographic guidance is associated with improved outcomes compared with a routine strategy. Our analysis also revealed the need for improved reporting standards regarding outcomes including mortality and HF-related hospitalization. Given the limited number of studies included in our analysis, the evidence for the improvement of clinical outcomes should be strengthened with data from additional clinical trials.

## Abbreviations

CRT: Cardiac resynchronization therapy
CRT-P: CRT pacemaker
CRT-D: CRT implantable cardioverter defibrillator
ECHO: Echocardiography
HF: Heart failure
LBBB: Left bundle branch block
LVEF: Left ventricular ejection fraction
LVESV: Left ventricular end-systolic volume
RCT: Randomized controlled trial
STE: Speckle-tracking echocardiography
VVI: Vector velocity imaging
